# New Cervical Cancer Screening Guidelines: Was the Annual Pap Too Much of a Good Thing?

**DOI:** 10.6004/jadpro.2013.4.1.7

**Published:** 2013-01-01

**Authors:** Margaret M. Fields

**Affiliations:** From MD Anderson Cancer Center, Houston, Texas

Fifty years ago in the United States, the leading cause of cancer death among women was cervical carcinoma. With the advent of the Papanicolaou (Pap) test in 1945, mortality from this malignancy declined more than 70% (Datta et al., 2008; Spitzer, 2007). An annual Pap smear was the recommended guideline for many years. New cervical cancer guidelines were released by the United States Preventative Services Task Force (USPSTF) on March 14, 2012.

## What Are the New Guidelines?

The following points summarize the new USPSTF cervical cancer screening guidelines:

- Begin screening at age 21 with use of cytology alone every 3 years from ages 21–29. No screening prior to age 21 regardless of age of coitarche.
- Patients with atypical squamous cells of undetermined significance (ASCUS) on cytology with negative human papillomavirus (HPV) status should be managed the same way as patients with negative cytology.
- Women ages 30–65 should be screened every 5 years with co-testing (cytology and HPV) or every 3 years with cytology alone if co-testing is not available.
- Women over age 65 can discontinue screening. Once discontinued, screening should not resume even if a woman has a new sexual partner.
- No screening is needed after hysterectomy if there is no prior history of cervical intraepithelial neoplasia grade 2 (CIN2) or higher grade lesion.
- Following spontaneous regression or treatment, women with a history of CIN2 or higher grade lesion should continue screening, even if this extends beyond age 65 (Schwaiger, Aruda, LaCoursiere, & Rubin, 2012).

## What Is Different From Past Guidelines?

The previous guidelines initiated screening 3 years after beginning sexual activity but no later than age 21. Women younger than 30 were recommended to have a Pap test every 2 years. For women 30 or older, the recommendation was every 3 years if they had had 3 consecutive negative Pap tests. These guidelines issued in 2009 recommended less frequent screening than the prior guidelines from 2003, which recommended women younger than age 30 have an annual exam (Solomon, Breen, & McNeel, 2008; Sawaya, 2009).

## Why the Change?

In order to understand why the previous screening guidelines, which resulted in such a dramatic decrease in mortality, have now changed, it is essential to understand how the knowledge of cervical carcinogenesis has progressed through the years.

Human papillomavirus is the primary causative agent of cervical carcinoma. Persistent infection is necessary for cancer to develop; this is a process that evolves over decades from preinvasive intraepithelial neoplasia to invasive cancer (Denny, 2012). Human papillomavirus is extremely prevalent; the lifetime risk of infection is 80% (Veldhuijzen, Snijders, Reiss, Meijer, & van de Wijgert, 2010). Current estimates are that 50% to 80% of young sexually active women will contract HPV infection within 24 to 36 months of coitarche (Datta et al., 2008). The prevalence of HPV infection in the US is 40% among sexually active women ages 14–19, and 49.3% among sexually active women 20–24 years old. The good news is that 90% of women have been found to clear the infection without intervention within 2 years. Human papillomavirus persistence, which is necessary for the development of cervical cancer, affects only a small percentage of women who acquire HPV (Veldhuijzen et al., 2010; Widdice & Moscicki, 2008).

The majority of cervical cancers are related to two specific strains of HPV. HPV 16 accounts for 51% of cervical cancers and HPV 18 for 16%. Additional strains of HPV that are related to cervical cancer include HPV 35 (8.7%) and HPV 45 (7.4%; Clifford, Smith, Plummer, Munoz, & Franceschi, 2003). This equates to a stronger correlation between high-risk HPV and cervical cancer than that between smoking status and lung cancer (Denny, 2012).

Young women are particularly prone to acquiring HPV infection due to the anatomy of the immature cervix. The adult cervix contains protective squamous epithelium, whereas the immature cervix contains columnar epithelium. During the adolescent period, this tissue undergoes metaplasia and transforms to squamous epithelium. During metaplasia, HPV has easier access to the basal cell layer, the site of rapid replication and differentiation (Hwang et al., 2012; Moscicki & Cox, 2010; Moscicki, 2007). Despite this susceptibility to HPV infection, 90% of young women are able to clear the infection without intervention and hence avoid development of invasive cervical cancer (Moore et al., 2010; Moscicki, 2007; Moscicki & Cox, 2010).

Women ages 15 through 19 have a very low incidence of invasive cervical cancer, at a rate of 0.1/100,000 (Moscicki & Cox, 2010). This rate has remained unchanged for 4 decades despite increased screening coverage (Castle & Carreon, 2010). Therefore, deferring the onset of cervical screening until age 21 is unlikely to have a significant impact on the overall incidence of cervical cancer. The benefit of delayed onset of screening is the reduction in the number of colposcopies and biopsies for young women with transient HPV changes that will likely resolve on their own in 6 to 36 months (Schwaiger et al., 2012; Moscicki & Cox, 2010). Not only is emotional and physical discomfort avoided, but there is a decrease in health-care costs seen with the elimination of unnecessary colposcopies and biopsies as well. Finally, there are long-term consequences of treatment of dysplasia with cryotherapy or the loop electrosurgical excision procedure (LEEP). These include cervical stenosis, incompetent cervix, increased risk of premature delivery, low birth weight, and premature rupture of membranes (Moscicki & Cox, 2010; Krygiou et al., 2006).

## Exceptions to the Guidelines

It is important to keep in mind that the new cervical cancer screening guidelines do not apply to women who are immunosuppressed, including patients with HIV or immunosuppression related to organ transplantation. Also excluded from these screening guidelines are women with a history of cervical cancer or in utero exposure to diethylstilbestrol (DES). Additional risk factors for the development of invasive cervical cancer to bear in mind include smoking and having a history of multiple sexual partners (Hwang et al., 2012; Schwaiger et al., 2012).

Surveillance, Epidemiology, and End Results (SEER) data demonstrate that cervical cancer is not a disease of youth and HPV acquisition, but rather one that occurs in the middle-aged to older female and is related to the persistence of HPV (National Cancer Institute, 2012); see Table 1. Based on what we have learned through the years about the cause of cervical cancer and the timeline for development from intraepithelial neoplasia to invasive cancer, the new guidelines are medically sound and should continue to adequately protect women from invasive disease.

**Table 1:**
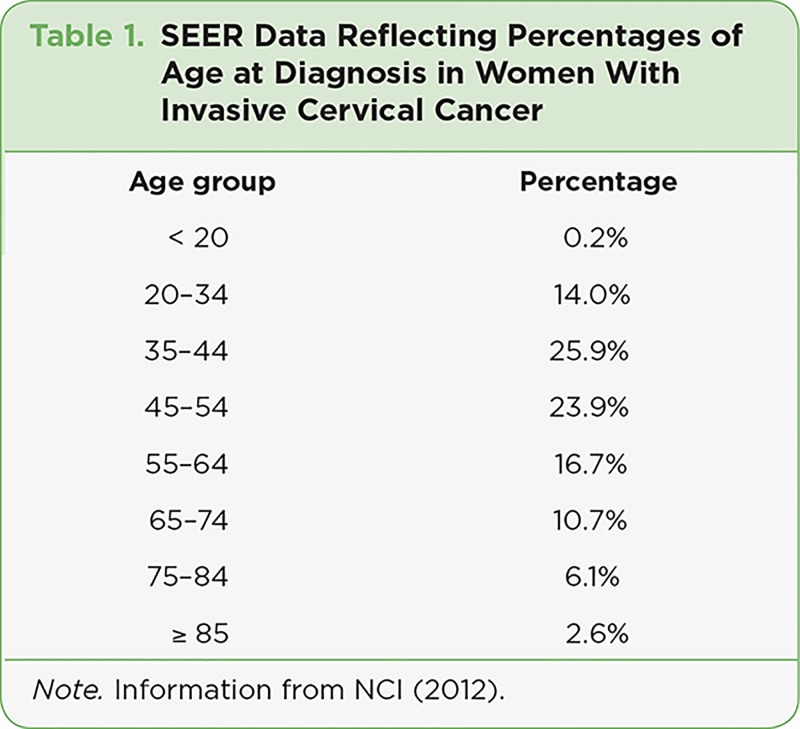
Table 1. SEER Data Reflecting Percentages of Age at Diagnosis in Women With Invasive Cervical Cancer

## Prevention

In recent years, we have learned more about the cause and development of invasive cervical cancer and are now poised to move from the practice of secondary prevention to primary prevention. In the past decade, vaccines have been developed to target high-risk strains of HPV. A bivalent vaccine (Cervarix [GlaxoSmithKline, 2009]) is targeted toward HPV 16 and 18. A quadravalent vaccine (Gardasil [Merck, 2006]) is targeted to HPV 6, 11, 16, and 18. Both are commercially available and approved for males and females ages 9 through 26. These vaccines are able to decrease the risk of cervical and other HPV-related cancers (Denny, 2012; Malagón et al., 2012).

## Conclusions

The focus of the well-woman examination may now shift from that of the annual Pap test to cancer screening, assessment of cardiovascular risk factors, and evaluation of thyroid health. The clinician will be better able to encourage fitness and promote overall health. As many young women do not regularly see a primary care physician, the annual well-woman visit may be their only health-care contact. Having more time to devote to issues of overall health and well-being will help build stronger relationships between clinicians and patients, resulting in increased mutual benefit.

## References

[1] Castle P. E., Carreon J. D. (2010). Practice improvement in cervical screening and management (PICSM): Symposium on management of cervical abnormalities in adolescents and young women. *Journal of Lower Genital Tract Disease*.

[2] Clifford G M, Smith J S, Plummer M, Muñoz N, Franceschi S (2003). Human papillomavirus types in invasive cervical cancer worldwide: a meta-analysis.. *British journal of cancer*.

[3] Datta S Deblina, Koutsky Laura A, Ratelle Sylvie, Unger Elizabeth R, Shlay Judith, McClain Tracie, Weaver Beth, Kerndt Peter, Zenilman Jonathan, Hagensee Michael, Suhr Cristen J, Weinstock Hillard (2008). Human papillomavirus infection and cervical cytology in women screened for cervical cancer in the United States, 2003-2005.. *Annals of internal medicine*.

[4] Denny Lynette (2012). Cervical cancer prevention: new opportunities for primary and secondary prevention in the 21st century.. *International journal of gynaecology and obstetrics: the official organ of the International Federation of Gynaecology and Obstetrics*.

[5] (2009). Cervarix package insert.. *GlaxoSmithKline*.

[6] Hwang Loris Y, Ma Yifei, Shiboski Stephen C, Farhat Sepideh, Jonte Janet, Moscicki Anna-Barbara (2012). Active squamous metaplasia of the cervical epithelium is associated with subsequent acquisition of human papillomavirus 16 infection among healthy young women.. *The Journal of infectious diseases*.

[7] Kyrgiou M, Koliopoulos G, Martin-Hirsch P, Arbyn M, Prendiville W, Paraskevaidis E (2006). Obstetric outcomes after conservative treatment for intraepithelial or early invasive cervical lesions: systematic review and meta-analysis.. *Lancet*.

[8] Malagón Talía, Drolet Mélanie, Boily Marie-Claude, Franco Eduardo L, Jit Mark, Brisson Jacques, Brisson Marc (2012). Cross-protective efficacy of two human papillomavirus vaccines: a systematic review and meta-analysis.. *The Lancet infectious diseases*.

[9] (2006). *Gardasil package insert*.

[10] Moore Gaea, Fetterman Barbara, Cox J Thomas, Poitras Nancy, Lorey Thomas, Kinney Walter, Castle Philip E (2010). Lessons from practice: risk of CIN 3 or cancer associated with an LSIL or HPV-positive ASC-US screening result in women aged 21 to 24.. *Journal of lower genital tract disease*.

[11] Moscicki Anna-Barbara (2007). HPV infections in adolescents.. *Disease markers*.

[12] Moscicki Anna-Barbara, Cox J Thomas (2010). Practice improvement in cervical screening and management (PICSM): symposium on management of cervical abnormalities in adolescents and young women.. *Journal of lower genital tract disease*.

[13] (2012). SEER Stat Fact Sheets: Cervix uteri. *National Cancer Institute*.

[14] Sawaya George F (2009). Cervical-cancer screening--new guidelines and the balance between benefits and harms.. *The New England journal of medicine*.

[15] Schwaiger Constance, Aruda Mary, LaCoursiere Sheryl, Rubin Richard (2012). Current guidelines for cervical cancer screening.. *Journal of the American Academy of Nurse Practitioners*.

[16] Solomon D., Breen N., McNeel T. (2007). Cervical cancer screening rates in the United States and the potential impact of implementation of screening guidelines. *CA: A Cancer Journal for Clinicians*.

[17] Spitzer Mark (2007). Screening and management of women and girls with human papillomavirus infection.. *Gynecologic oncology*.

[18] Veldhuijzen Nienke J, Snijders Peter Jf, Reiss Peter, Meijer Chris Jlm, van de Wijgert Janneke Hhm (2010). Factors affecting transmission of mucosal human papillomavirus.. *The Lancet infectious diseases*.

[19] Widdice Lea E, Moscicki Anna-Barbara (2008). Updated guidelines for papanicolaou tests, colposcopy, and human papillomavirus testing in adolescents.. *The Journal of adolescent health : official publication of the Society for Adolescent Medicine*.

